# The improvement and verification of fluid dynamics simulation on temperature uniformity during heat treatment of ring pieces

**DOI:** 10.1016/j.heliyon.2024.e36099

**Published:** 2024-08-13

**Authors:** Mingzhe Xu, Jinfu Zhao, Li Wang, Tengxiang Zhao, Ling Kong, Zhipeng Li, Zhixin Huang, Yuhui Wang

**Affiliations:** a National Engineering Research Center for Equipment and Technology of Cold Strip Rolling, Yanshan University, Qinhuangdao, 066004, China; b Chongqing Industry Polytechnic College, Chongqing, 401020, China; c Institute of Flexible Electronics, Northwestern Polytechnical University, Xian, 710072, China; d Pera Corporation Ltd, Beijing, 100025, China

**Keywords:** Fluid dynamics simulation, Heat treatment, Temperature field, Fluid field, Desktop heat treatment furnace

## Abstract

The improvement and verification of fluid dynamics simulation on temperature uniformity during the heat treatment of ring pieces are investigated in this study. The accuracy of the temperature field model is validated by comparing the simulation results with the measured temperatures. The findings reveal that the vortex generated near the furnace wall during heat treatment significantly affects the uniformity of the temperature field. To improve this, adjustments are made to the placement of ring pieces based on an experimentally validated fluid dynamics simulation model, and subsequent calculations are performed on this adjusted model. It is observed that these adjustments greatly enhance temperature uniformity in the heating process, with a 39.06 % improvement in medium-temperature zone (732.32–743.69 k) within the furnace compared to the original model. Additionally, surface temperatures of ring pieces in another medium-temperature zone (668.89–691.11 k) show a 34.54 % improvement in comparison to those predicted by the original model.

## 1 Introduction

In response to the severe impacts of climate change, major global economies have gradually developed carbon reduction policies to address this issue. On December 11th, European Union introduced the European Green New Deal, which sets a target of reducing emissions to 50 % of the 1990 level by 2030 and further striving for additional reductions. Additionally, the deal aims to achieve carbon neutrality by 2050 and establish legal measures to promote its realization. China has also implemented a series of policies with the goal of achieving carbon neutrality by 2060 after reaching a peak in carbon emissions by 2030. In the equipment manufacturing industry, heat treatment requires significant electricity consumption. Typically, heat treatment accounts for approximately 25 %–30 % of total electricity consumption within enterprises. In China's electric heating device-related heat treatment production capacity in 2014 was recorded at around 13.5 million tons; however, actual annual power consumption reached10 billion kw·h [1].Sinosteel Xingtai Mechanical Rolling Co., Ltd., for instance, has an annual production capacity of 180,000 tons for rolls and 80,000 tons for metallurgical equipment with a total annual power consumption estimated at approximately 0.064 billion kw·h.

The energy consumption in the heat treatment process of ring pieces at China Steel Xingtai Machinery Roll Co., Ltd. will be reduced by optimizing the placement mode through fluid dynamics simulation. The ring pieces are made of Cr12MoV material, which is widely used in cold work mold steel [2,3]. Cr12MoV has a carbon content of 1.45–1.70 % and a chromium content of 11.50–13.00 %. Due to its high carbon and chromium content, it forms a large amount of chromium carbide and high-alloyed martensite, resulting in high hardness and wear resistance. Mo and V are also added to improve impact toughness [4,5]. Therefore, Cr12MoV is commonly used for manufacturing stamping and drawing dies [6]. When making cold-drawing dies, Cr12MoV material needs to undergo heat treatment to achieve dynamic recrystallization. Dynamic recrystallization can refine and homogenize the structure of Cr12MoV, eliminating work-hardening phenomenon and internal stress while reducing hardness. The study conducted by Sun on hot compression of Cr12MoV die steel, however, revealed that the dynamic recrystallization (DRX) behavior exhibited temperature sensitivity [7], so precise temperature control during the heat treatment process of Cr12MoV is crucial for research purposes. The traditional chamber heat treatment furnace cannot accurately control the heating and cooling temperatures of the workpiece, leading to poor uniformity in solid phase transformation of metal materials as well as increased internal stress and Causes plastic deformation.

Michalcová has discovered that severe plastic deformation significantly impacts the structure and performance of products [8]. Furthermore, Milanese has identified that specific material structures or external factors can have adverse effects on the properties of metallic materials [9], manual regulation cannot ensure balanced internal temperature within metal materials effectively. Improper thermal treatment can result in waste of materials and energy consumption while generating a significant amount of CO_2_ emissions.

The Fluent software is utilized in this study to conduct simulations of the temperature and fluid fields involved in heating the ring pieces within a benchtop furnace [10,11]. Accurate simulation results are obtained for the temperatures at various locations on the surface of the ring pieces during the heating process. These simulation results are then compared with experimental measurements to validate the accuracy of our simulation model. The fabrication method of Cr12MoV ring pieces is re-improved based on the numerical model and the relationship between the fluid flow field and the temperature field [12,13]. Subsequently, a new improved model is established [14] and simulated, which confirms its feasibility in improving the temperature uniformity of the ring pieces.

## 2 Model and methods

### 2.1 Benchtop heat treatment furnace modeling

**Fig. 1:**
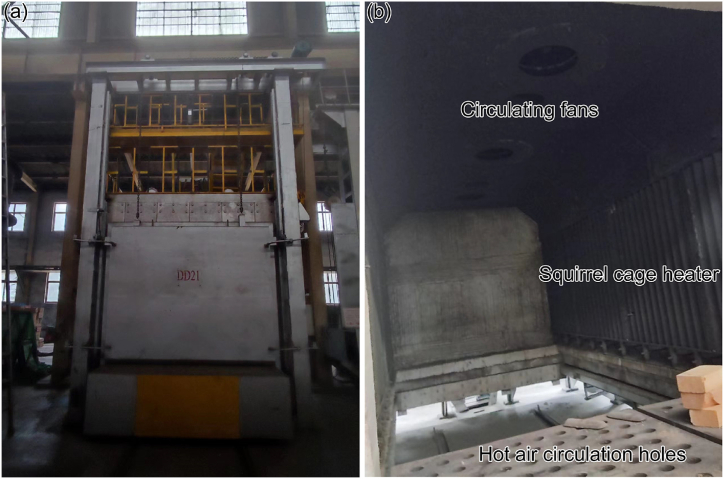
DD21 furnaces: (a) the front view and (b) the internal view.

**Fig. 2:**
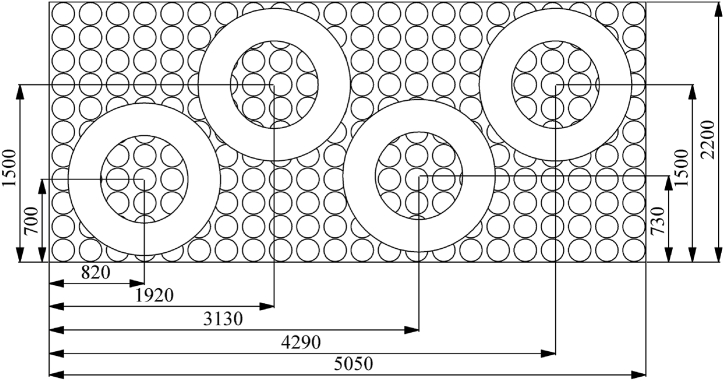
Engineering drawings of ring piece placement.

The research object of this paper is the heating process of ring pieces in the heat treatment workshop of China Steel Xingtai Machinery Roll Co., Ltd [15,16]. The structure of a benchtop heat treatment furnace mainly comprises a furnace shell, inner liner, squirrel cage heater, circulating fan, and heating ring pieces. The squirrel cage heater is positioned inside the side wall. The tabletop furnace is divided into three heating zones; each zone has 12 squirrel cage heaters, and the lower hole is the hot air circulation hole (Fig. 1(a and b)). Fig. 2 shows the ring piece's placement engineering diagram (unit: mm). There are 16 ring pieces in the tabletop heat treatment furnace placed in 4 stacks; each stack comprises four layers, and clay bricks separate each ring piece.

### 2.2 Meshing

**Fig. 3:**
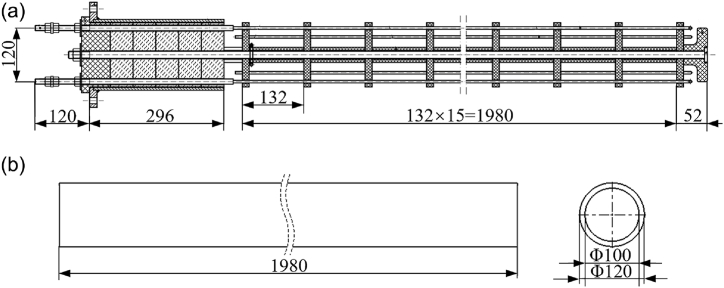
The dimensional drawing of the heater: (a) the original structure and (b) the simplified structure.

The complex furnace structure will increase the number of mesh elements, reduce their quality, and take up more computational and memory resources. Therefore, the following simplifications are made to the original furnace structure. First, the circulating fan structure of the original physical model is simplified. The original model of the circulating fan is replaced by the circular face without thickness and the pressure difference between the two sides. Secondly, the squirrel cage heater is simplified by using a hollow cylinder with the same heating volume. The squirrel cage heater is shown in Fig. 3. Fig. 3(a) shows the physical model diagram, and Fig. 3(b) shows the simplified model diagram (unit: mm). Finally, the benchtop furnace insulation layer is simplified, where a two-dimensional heat transfer wall replaces the insulation layer of the heat treatment furnace. The insulation layer mainly dissipates heat to the outside world by radiation and convection [17,18]. The multilayer integrated thermal conductivity is used, and the integrated thermal conductivity is calculated as follows [19]:

(1) $$\lambda =\frac{1}{\frac{b_{1}}{\lambda_{1}b}+\frac{b_{2}}{\lambda_{2}b}+\frac{b_{3}}{\lambda_{3}b}}$$

where *b*_*1*_, *b*_*2*_, *b*_*3*_ …… are the thicknesses of each insulation layer, *b* is the thickness of the entire insulation layer, λ_*1*_, *λ*_*2*_, *λ*_*3*_ ……*λ*_*i*_ are the thermal conductivities of each layer, and *λ* is the integrated thermal conductivity.

**Fig. 4:**
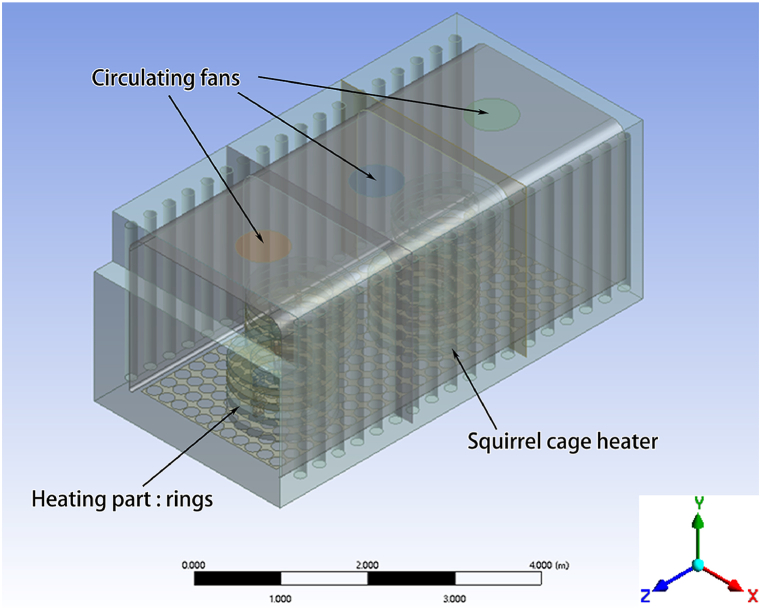
The model diagram of the benchtop heat treatment furnace.

After simplifying the original model of the circulating fans, squirrel cage heater, and insulation layer, the final model diagram of the benchtop heat treatment furnace is obtained and shown in Fig. 4.

### 2.3 Mesh division

**Fig. 5:**
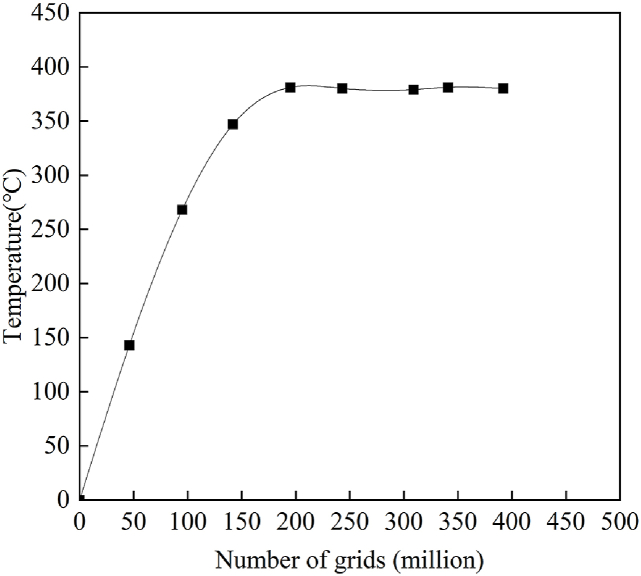
Mesh-independent verification.

**Fig. 6:**
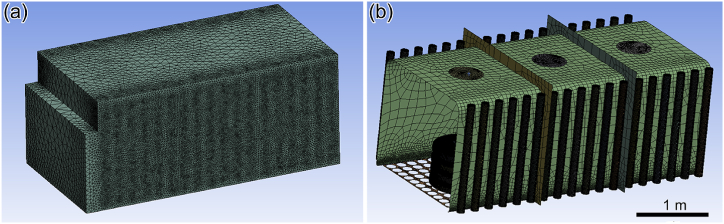
Mesh delineation of the heat treatment furnace: (a) the overall mesh and (b) the locally encrypted meshing.

The mesh is mainly used to characterize the simulation model's geometric structure and solution domain. The number of mesh elements is not increased since an overly dense mesh consumes substantial computational and memory resources. In order to select the appropriate number of grids, the grid-independence verification of the simulation model is carried out, and the verification results are shown in Fig. 5. From Fig. 5, it can be concluded that when the number of grids is larger than 2.17 million, the neighboring solutions are basically consistent. The heat treatment furnace model is meshed using the tetrahedral mesh method with a cell size of 1. Finally, a mesh model with 2,417,721 elements and an orthogonal mass of 0.226677 is generated. Fig. 6 shows the results of the heat treatment furnace model delineation diagram. Fig. 6(a) depicts the tetrahedral division of the mesh map and Fig. 6(b) depicts the mesh encryption map with the squirrel cage heater and the ring sheet.

### 2.4 Mathematical model

During the heat treatment of ring pieces, heat generation and transfer occurs. In this process, due to the uneven furnace temperature roller ring may occur plastic deformation, at the same time roller ring internal stress size and distribution will also change with the distribution of the temperature field. Therefore, the nonlinear factors in the temperature field, such as the integrated heat transfer coefficient, the thermal and physical parameters of the material, and the latent heat of phase transition, must be accurately grasped in the numerical calculations. For the three-dimensional nonlinear steady state heat transfer problem, the mathematical expression for the heat transfer equation is as follows [20]:

(2) $$\frac{\partial }{\partial x}\left(k_{x}\frac{\partial T}{\partial x}\right)+\frac{\partial }{\partial y}\left(k_{y}\frac{\partial T}{\partial y}\right)+\frac{\partial }{\partial z}\left(k_{z}\frac{\partial T}{\partial z}\right)+q=0$$

where T is the temperature, *k*_*x*_, *k*_*y*_, *k*_*z*_ are the thermal conductivity of the material in the *x, y, z* directions, *q* is the internal heat source term. The mathematical expression for the boundary conditions is as follows [21]:

(3) $$k_{x}\frac{\partial T}{\partial x}n_{x}+k_{y}\frac{\partial T}{\partial y}n_{y}+k_{z}\frac{\partial T}{\partial z}n_{z}-q+h\sum \left(T-T\infty \right)=0$$

where *h∑* is the combined heat transfer coefficient of convection and radiation, *q* is the heat flow term; *n*_*x*_, *n*_*y*_, *n*_*z*_ are the directional cosines in the *x*, *y* and *z* directions, respectively.

### 2.5 Parameter settings

The gas inside the furnace is assumed to be incompressible, and the calculation method adopts the SIMPLE algorithm, radiation model selection S2S. Moreover, the turbulence model [22,23] ere adopts the standard k-ε model to improve the external flow problem around the complex geometry [24]. According to the parameters provided by the manufacturer, the material of the ring pieces is set as Cr12MoV, the specific components are shown in Table 1, the density is set as 7.85 g/cm^3^. The material of the squirrel cage heater is a nickel-chromium alloy. The density is set to 8.42 g/cm³, the specific heat capacity to 460 J/(kg·k), and the heat conduction coefficient to 60.3 w/(m·k) (at 500 °C).

**Table 1:** Chemical composition of the Cr12MoV steel (mass fraction,%).

C	Si	Mn	P	S	Cr	Ni	Cu	Mo	V	Fe
1.53	0.32	0.38	0.024	0.003	11.38	0.19	0.09	0.52	0.25	Bal.

According to the control system of the heat treatment furnace, the air pressure of the circulating fan is set at 1200Pa. The simulation of the circulating fan wind is achieved by utilizing a pressure difference, with both sides of the fan having a pressure difference of 1200 Pa. This condition serves as boundary conditions for the numerical model of the fan. Additionally, power curves are derived for each heating zone during the warming process. Since there is minimal variation in power among these zones during warming, an average value is chosen and used as thermal boundary conditions for the numerical model. These values are presented in Table 2, representing average power recorded over 2 h in three heating zones during benchtop furnace heating process, along with calculated volumetric power values.

**Table 2:** The average power value of the three heating zones with the calculated volumetric power values.

Power/Volume power	Inner area	Middle area	External area
Power (w)	6.6 × 10^4^	4.9 × 10^4^	6.3 × 10^4^
Volume power (w/m^3^)	9.7 × 10^6^	7.2 × 10^6^	9.3 × 10^6^

* The heating volume of each group of squirrel cage heaters is 6.84 × 10^-^³ m³.

**Fig. 7:**
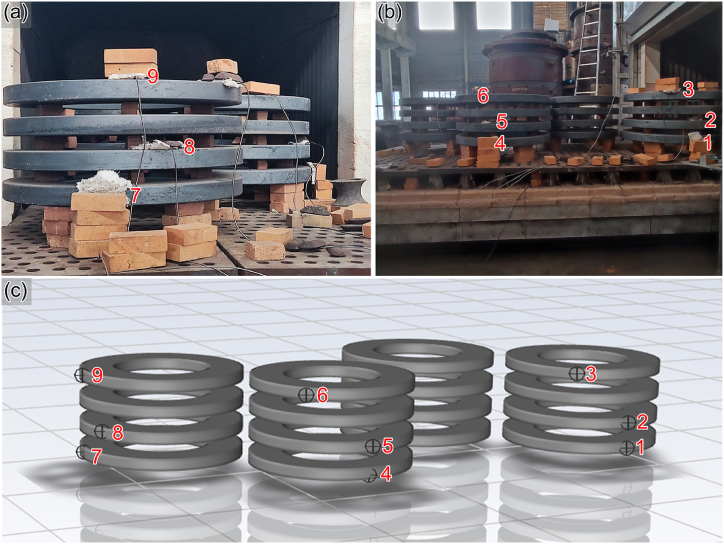
Schematic diagrams of the thermocouple arrangement in the field and the model: (a) and (b) the location of the thermocouples in the field, (c) the location of the thermocouples.

The initial temperature of the furnace is set to 350 °C according to the initial temperature measured by the thermocouple. The solution mode is set to a transient solution, the maximum incremental step is 144 00 steps, and the incremental step size is set to 0.5. The ring pieces are equipped with nine thermocouple thermometers to monitor the changes of temperature. A coordinate system is established within the heat treatment furnace to determine the precise coordinates of each thermocouple. Additionally, simulated temperature values are generated at specific coordinate points during the simulation process to validate the accuracy of the model. Fig. 7(a) and (b) represent the thermocouple field arrangement diagrams, while Fig. 7(c) represents the schematic diagram of the model temperature output points. The serial numbers from 1 to 9 represent the thermocouple arrangement positions.

## 3 Results and discussion

### 3.1 Comparative analysis of simulated and measured results

**Fig. 8:**
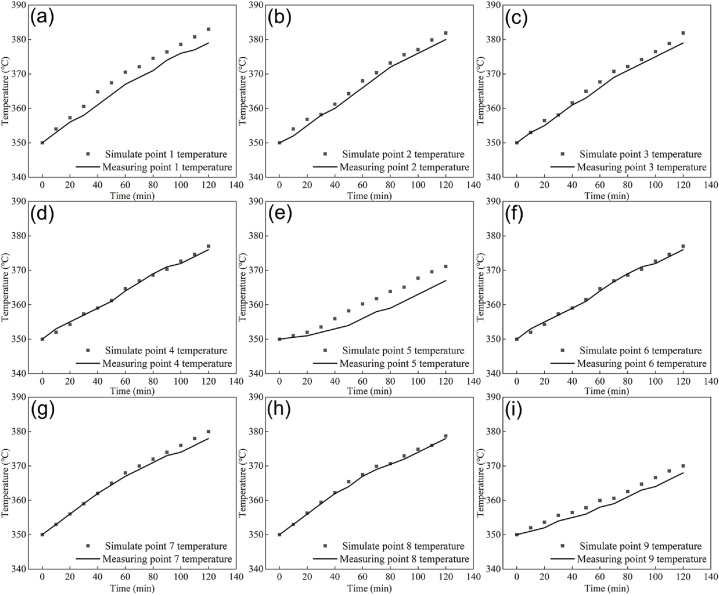
The simulated temperature of the ring pieces heating stage versus the measurement points: (a)–(i) represent nine temperature measurement points.

The heat treatment of ring pieces is divided into three stages: heating, holding, and cooling. The selection of the heating process has the greatest impact on the microstructure of workpieces and mechanical properties after heat treatment. However, an excessively rapid heating rate will result in grain coarsening and even direct cracking. Therefore, precise control over the heating rate is necessary. Fig. 8 compares the simulated and measured point temperatures during the heating phase of the ring pieces. The scatter plot represents the simulated temperature, while the curve represents the measured temperature. According to Fig. 8(a–i), points 1, 2, and 3 in the inner area of the furnace exhibit higher temperatures in both simulated and measured values. Similarly, points 7, 8, and 9 in the outer area show lower temperatures as expected from actual temperature variations within the furnace. The trend of measured and simulated temperatures at these nine points aligns with each other. Among them, point 1 records highest temperature measurement with a simulated value reaching up to 383 °C which deviates by only 3 °C from actual measurements. On contrary, point 9 records lowest temperature measurement with a highest simulated value being at 370 °C which differs by 13 °C from actual measurements. Comparing all these data reveals that after 2 h of temperature increase, the largest error occurs at point 5, i.e., 1.57 %. However, the errors for remaining simulated and measured temperature points are within the allowable error range of less than 1.57 %. Therefore, it can be concluded that the simulation model construction is accurate [25,26].

### 3.2 Distribution of temperature and fluid fields within a benchtop heat treatment furnace model

The microstructure and macro-mechanical properties of ring pieces are influenced by the heat treatment process. Incomplete phase transformation, uneven distribution of tissue composition, and increased tissue stress due to the overall temperature difference in the ring pieces can ultimately lead to deformation of the workpiece. Therefore, it is essential to monitor and maintain real-time overall temperature uniformity during the heating process of ring pieces.

**Fig. 9:**
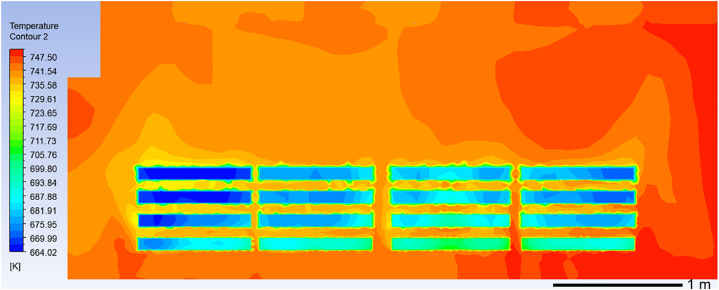
Temperature field distribution in the furnace chamber after 7200 s.

Fig. 9 illustrates a simulated cloud diagram depicting the temperature field in the YZ section when ring pieces are heated in a benchtop heat treatment furnace for 120 min. According to Fig. 9, there is relatively uniform temperature distribution throughout the entire internal cavity of the furnace with an overall temperature difference of approximately 30 °C. The inner and middle areas exhibit consistent temperature differences ranging from a maximum of around 12 °C to a minimum of about 5 °C. However, there is a larger overall temperature difference in the outer area with a maximum difference reaching approximately 30 °C. The slow gas flow rate and low temperature of the gas on the surface of the low-temperature part of the ring pieces in the outer zone result in inefficient convective heat transfer with the ring pieces, leading to a lower temperature in this area. Conversely, near the bottom of the heat treatment furnace, where there is a fast gas flow rate and high temperature, convective heat transfer efficiency is high and results in higher temperatures for the ring pieces. This creates a significant temperature difference between the outer zone and yz cross-section.

**Fig. 10:**
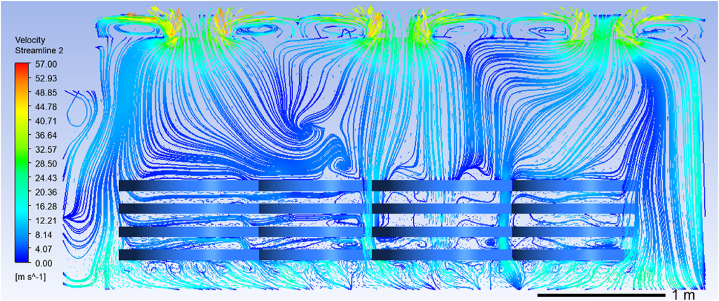
Distribution of flow velocity field in the furnace chamber during heating.

Similarly, simulation results also provide insights into fluid dynamics within the benchtop heat treatment furnace during ring piece heating (Fig. 10). Air inside the furnace is drawn through circulating fan ports, heated by heaters, and then circulated into the furnace through hot air circulation holes located at its bottom for heating purposes on ring pieces. Moreover, gas flow rates are higher in both inner and middle areas compared to those observed in outer regions. In particular, within inner areas where gas flows through cavities between ring pieces, maximum flow rate reaches approximately 35 m/s while slowest flow rate occurs on walls above these inner areas at roughly 15 m/s on the wall of the ring pieces above the inner area. The overall gas flow rate in the outer area is slow. The flow rate through the lower ring pieces is approximately 18 m/s, and the flow rate through the upper ring pieces is nearly 5 m/s. The tabletop furnace also produces large and small vortices in the inner zone of the cycle, and the vortex phenomenon is more obvious in the outer zone.

### 3.3 Analysis of the coupling relationship between temperature and fluid fields

**Fig. 11:**
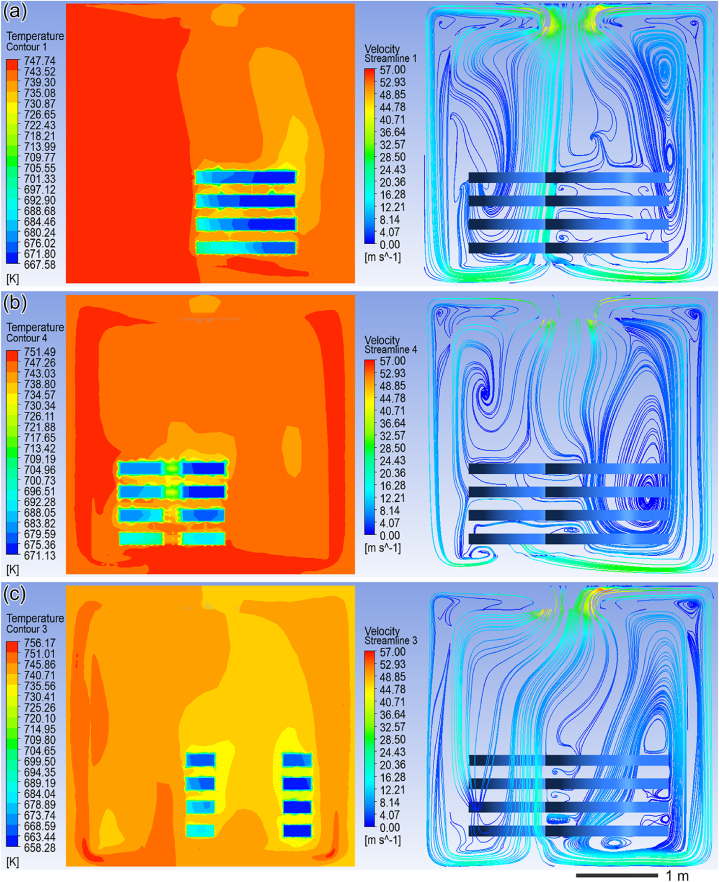
Cloud diagram of temperature field fluid field in the XY section: (a) the outer zone, (b) the middle zone, and (c) the inner zone.

The temperature and fluid fields of the three XY sections in the outer (Fig. 11(a)), middle (Fig. 11(b)), and inner areas (Fig. 11(c)) were captured at 120 min of heating for studying and analyzing the correlation between the temperature field and fluid field of the benchtop furnace, as depicted in Fig. 11. Based on Fig. 11, a significant vortex is formed in the upper right corner of the XY section within the outer area. However, in the middle-left region, air flows directly from hot air circulation holes into the circulation fan through ring pieces. The temperature field maps indicate that this vortex causes gradual warming of air in this region, subsequently leading to a slow increase in vortex temperature.

The fluid field map of XY section within the middle area displays a large vortex on its right side. When combined with temperature field map of XY section within this zone, it reveals a low-temperature zone at center of this vortex. Additionally observed is that gas flow rate is higher at edge regions of this vortex resulting in faster warming up of ring pieces located there. According to fluid field maps for XY section within inner area, gas flow rate through hot air circulation holes positioned along axis is faster compared to those farther away from axis. Combining these observations with temperature field maps for XY section within inner zone indicates that ring piece temperatures are highest near axis and decrease as they move away from it.

### 3.4 Improvement model simulation experimental analysis

**Fig. 12:**
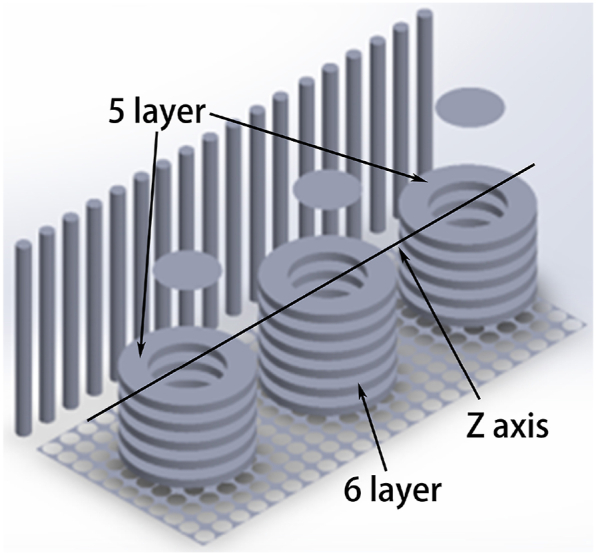
Model map of ring pieces placement to the Z-axis.

Analysis regarding relationship between temperature and fluid fields during heat treatment process reveals that eddy currents present in fluid fields impact uniform distribution of furnace temperatures while uniformity inside furnace affects heat treatment process's overall temperature consistency for ring pieces being treated. For same vortex pattern observed, flow rate at edges tends to be faster while central flow rates are slower, while the temperature in the center of the vortex is slightly lower than at the edge. According to Fig. 11, it can be concluded that the area directly under the circulating fan has a faster gas flow rate and is less likely to produce vortices. Therefore, another set of improvement simulation experiment is set up. The ring pieces are placed on the Z-axis below the circulating fan of the bench-top furnace in 5, 6, and 5-layer combinations, as shown in Fig. 12. Lastly, it is ensured that other boundary conditions remain constant.

**Fig. 13:**
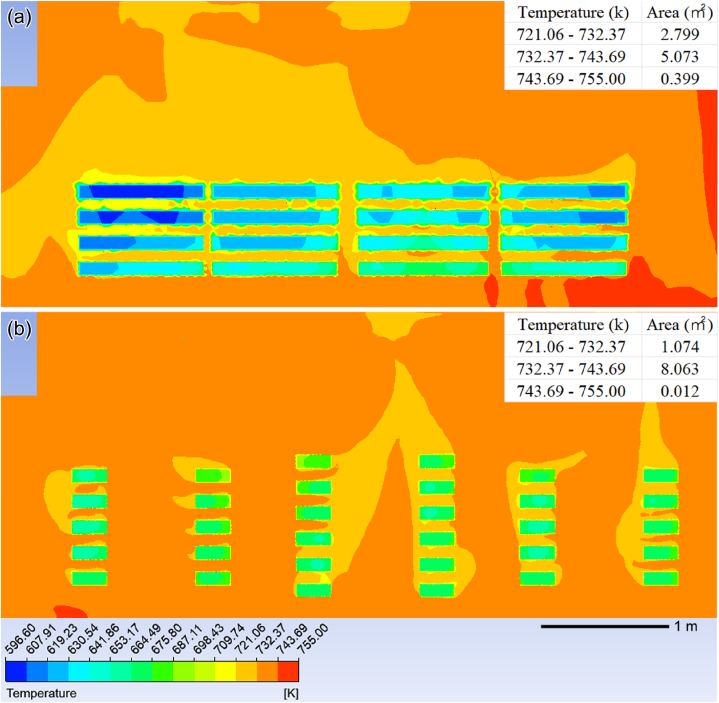
Comparison between the original model and the improved model in the YZ section temperature field clouds: the temperature cloud of yz section of the original model (a) and the temperature cloud of yz section of the improved model (b).

The temperature field clouds of the improved model and the original model at 659.6–775.0 k are compared to investigate the impact of ring pieces' placement on temperature uniformity in the furnace. Fig. 13(a) illustrates the temperature field cloud diagram of the YZ section in the original model, while Fig. 13(b) presents the temperature field cloud diagram of the YZ section in the improved model. The analysis of furnace interior temperature (excluding ring pieces) within a range of 721.06–755.00 k can be conducted based on these diagrams. The temperature ranges from 721.06 to 755.00 k is divided into three phases for quantitative analysis of temperature field uniformity in the YZ-section: low-temperature zone (721.06–732.37 k), medium-temperature zone (732.37–743 0.69 k), and high-temperature zone (743 0.69–755 0.00 k). As shown in Fig. 13, the areas of the low-temperature, medium-temperature, and high-temperature zones were calculated from ImageJ. Then, the proportion of each temperature zone to the total temperature cloud map is calculated and plotted in Table 3. Table 3 shows calculated percentage values for each temperature region in the YZ section.

**Table 3:** Comparison of temperature field uniformity between the original model and the improved model in the YZ section.

Original/Improved	721.06–732.37 k	732.37–743.69 k	743.69–755.00 k
Original model share	33.84 %	61.33 %	4.82 %
Improved model share	11.74 %	88.13 %	0.13 %

According to Table 3, there is a reduction of approximately 22.1 % in low-temperature zone for the improved model compared to the original one. The medium-temperature zone in the improved model increases by 26.8 %, while the high temperature zone experiences a 4.69 % decrease compared to the original model. It can be concluded that the temperature field uniformity in the furnace is greatly improved during the ring pieces heat treatment process in the improved model.

**Fig. 14:**
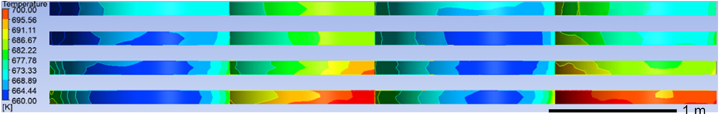
Temperature field cloud of the original model ring pieces.

**Fig. 15:**
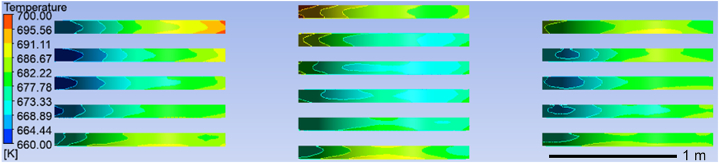
Improved model ring pieces temperature field.

To examine how ring pieces' placement affects surface temperature uniformity, we compare the distribution of temperatures between ring pieces of the improved model and those of the original model at 660–700 k. Fig. 14 depicts the temperature field cloud for the ring pieces in the original model, and Fig. 15 shows the temperature field diagram for the improved model's ring pieces. Similarly, the surface temperature range of the ring pieces from 660 k to 700 k is divided into three temperature stages, i.e., 660.00–668.89 k in the low-temperature region, 668.89–691.11 k in the medium temperature region, and 691.11–700 k in the high-temperature region. As shown in Table 4, the area of the surface low-temperature zone medium-temperature zone high-temperature zone for each stack of ring pieces was also calculated using ImageJ. Finally, the percentage values of the surface temperature intervals of the ring pieces were calculated by, and the results are shown in Table 5.

**Table 4:** Statistics of surface temperature distribution of rings per stack for the original and improved models.

	original model				improved Models		
temperature range	Stack 1	Stack 2	Stack 3	Stack 4	Stack 1	Stack 2	Stack 3
600.00–668.89 k	2.74 %	9.34 %	4.45 %	17.69 %	2.03 %	2.24 %	2.51 %
668.89–691.11 k	15.32 %	14.19 %	15.61 %	11.63 %	25.56 %	31.04 %	34.69 %
691.11–700.00 k	3.43 %	2.08 %	2.89 %	0.63 %	0.81 %	0.64 %	0.48 %

**Table 5:** Comparison of surface temperature field uniformity between the original model of the YZ segment and the improved model ring piece.

Original/Improved	600.00–668.89 k	668.89–691.11 k	691.11–700.00 k
Original model share	34.22 %	56.75 %	9.03 %
Improved model share	6.78 %	91.28 %	1.94 %

According to Table 4, the percentage distribution of roll rings in each temperature interval for both the original and modified models can be obtained, which is further simplified as shown in Table 5.

According to Table 5, the improved model's low-temperature zone on the ring pieces surface is reduced by 27.44 % compared with the original model. Furthermore, the medium-temperature zone on the ring piece surface of the improved model is increased by 34.53 %, and the high-temperature zone on the ring piece surface of the improved model is reduced by 7.09 % compared with the original model. It can be concluded that the temperature uniformity of the ring pieces surface of the improved model is greatly improved in the heat treatment process.

The establishment of a suitable experimental environment in our laboratory will be undertaken in the future to measure the temperature of the core of the ring pieces, thereby further validating the accuracy of the model.

## 4 Conclusions

By conducting an analysis of the correlation between the fluid flow field and temperature distribution within the heat treatment furnace during the heating process of ring pieces, it has been observed that both vortex formation within the furnace and gas flow rate on the surface of the ring pieces significantly impact temperature uniformity. Specifically, lower temperatures are recorded in the central region of a given vortex compared to its peripheral area, while decreased gas flow rates through the ring piece surface result in reduced temperatures within that specific region.

Based on the existing findings for resetting the fabrication method of the ring pieces, it was observed in the simulation of the improved numerical model that compared to the original model, there was a reduction of 32.53 % in the low temperature zone within the yz cross-section temperature map in the improved model; an increase of 39.06 % in the mid-temperature zone; and a decrease of 3.4 % in the high temperature zone. In terms of roller ring surface temperature cloud, there was a decrease of 27.44 % in the low temperature zone, an increase of 34.54 % in the medium temperature zone, and a decrease of 7.09 % in high temperature zone. These results validate that implementing improvements to heat treatment process effectively enhances uniformity across temperatures for ring pieces.

## Funding

The research was funded by 10.13039/501100012166National Key Research and Development Program (2022YFB3705500), 10.13039/501100015286Key Research and Development Program of Hebei Province (21310301D), Central Guide Local Science and Technology Development Fund Funded Projects (226Z1003G) and Province Natural Science Foundation Innovation Group Funding Project of Hebei (E2021203011).

## Data availability

Data associated with the study has not been deposited into a publicly available repository. Data will be available from the author on reasonable request.

## CRediT authorship contribution statement

**Mingzhe Xu:** Software, Methodology, Investigation, Formal analysis, Data curation. **Jinfu Zhao:** Software, Methodology, Investigation, Formal analysis, Data curation. **Li Wang:** Methodology, Investigation, Data curation. **Tengxiang Zhao:** Methodology, Formal analysis, Data curation. **Ling Kong:** Visualization, Validation, Resources, Conceptualization. **Zhipeng Li:** Writing – original draft, Methodology, Investigation, Conceptualization. **Zhixin Huang:** Writing – review & editing, Writing – original draft, Supervision, Software, Resources, Data curation. **Yuhui Wang:** Writing – review & editing, Writing – original draft, Supervision, Resources, Project administration, Funding acquisition.

## Declaration of competing interest

The authors declare that they have no known competing financial interests or personal relationships that could have appeared to influence the work reported in this paper.
